# Interplay between genetic, epigenetic, and gene expression variability: Considering complexity in evolvability

**DOI:** 10.1111/eva.13204

**Published:** 2021-02-19

**Authors:** Jean‐Pascal Capp

**Affiliations:** ^1^ Toulouse Biotechnology Institute INSA CNRS INRAE University of Toulouse Toulouse France

**Keywords:** cancer evolution, cell‐to‐cell heterogeneity, chromatin, DNA repair, stochastic gene expression

## Abstract

Genetic variability, epigenetic variability, and gene expression variability (noise) are generally considered independently in their relationship with phenotypic variation. However, they appear to be intrinsically interconnected and influence it in combination. The study of the interplay between genetic and epigenetic variability has the longest history. This article rather considers the introduction of gene expression variability in its relationships with the two others and reviews for the first time experimental evidences over the four relationships connected to gene expression noise. They show how introducing this third source of variability complicates the way of thinking evolvability and the emergence of biological novelty. Finally, cancer cells are proposed to be an ideal model to decipher the dynamic interplay between genetic, epigenetic, and gene expression variability when one of them is either experimentally increased or therapeutically targeted. This interplay is also discussed in an evolutionary perspective in the context of cancer cell drug resistance.

## 1 INTRODUCTION

The major technological breakthroughs made during the last 20 years allowed the analysis of many biological processes at the single‐cell level and revolutionized molecular biology. Molecular and cellular processes do not occur deterministically. A precise molecular process can exhibit a great variability in the sequence of its steps and in its final products that generates a very large degree of intercellular and intermolecular heterogeneity.

Especially, results obtained on individual cells and molecules have invalidated the deterministic view of gene expression. As the reactions governing gene expression involve a small number of molecules, it was previously assumed that they may exhibit random fluctuations, a phenomenon often called gene expression noise (McAdams & Arkin, 1999). These stochastic fluctuations in gene expression started to be finely quantified in the early 2000s when the rate of protein synthesis was measured in genetically identical cells placed in a homogeneous environment (Blake et al., 2003; Elowitz et al., 2002; Ozbudak et al., 2002; Raser & O'Shea, 2004). The role of molecular interactions within chromatin has been emphasized in the generation of this variability. In particular, regulatory proteins associate probabilistically with chromatin and produce cell‐to‐cell variability in gene expression (Voss et al., 2009). Also, evolution due to selection arises through variability and heritability of fitness‐related features, and feedback regulations were shown to be important modulators for both variability and phenotypic heritability. Positive and negative feedback regulation can affect the level of variability, and positive feedback can preserve phenotypic states over many cell generations (Becskei et al., 2001; Becskei & Serrano, 2000; Nevozhay et al., 2009, 2012).

Different sources of gene expression variability have been distinguished: The intrinsic (allele‐specific) variability is actually related to the random protein binding events on the gene or mRNA regulatory regions, while the extrinsic variability refers to fluctuations that originate from sources affecting multiple alleles, thus related to the underlying cell state and metabolism of each cell (Elowitz et al., 2002) (e.g., different number of ribosomes or RNA polymerases, different cell size or cell cycle stage, spatial (micro‐)environmental variability…) [see (Foreman & Wollman, 2020) for a recent demonstration of the major role of the extrinsic component in mammalian cells]. Thus, the expression level of a protein in a cell population reflects the probability of gene expression in each cell (Kaern et al., 2005). Changes in the mean expression level in the population are the consequence of changes in the probability of its expression in each cell.

All the major fields of molecular and cellular biology are now aware of the necessity to take into account this source of nongenetic heterogeneity: This important contributor to phenotypic diversification can have important consequences from developmental to evolutionary processes (Ackermann, 2015; Eling et al., 2019; Raj & van Oudenaarden, 2008). However, its relationships with other processes that produce phenotypic variation by acting at the genetic or epigenetic level are only partly deciphered.

**FIGURE 1:**
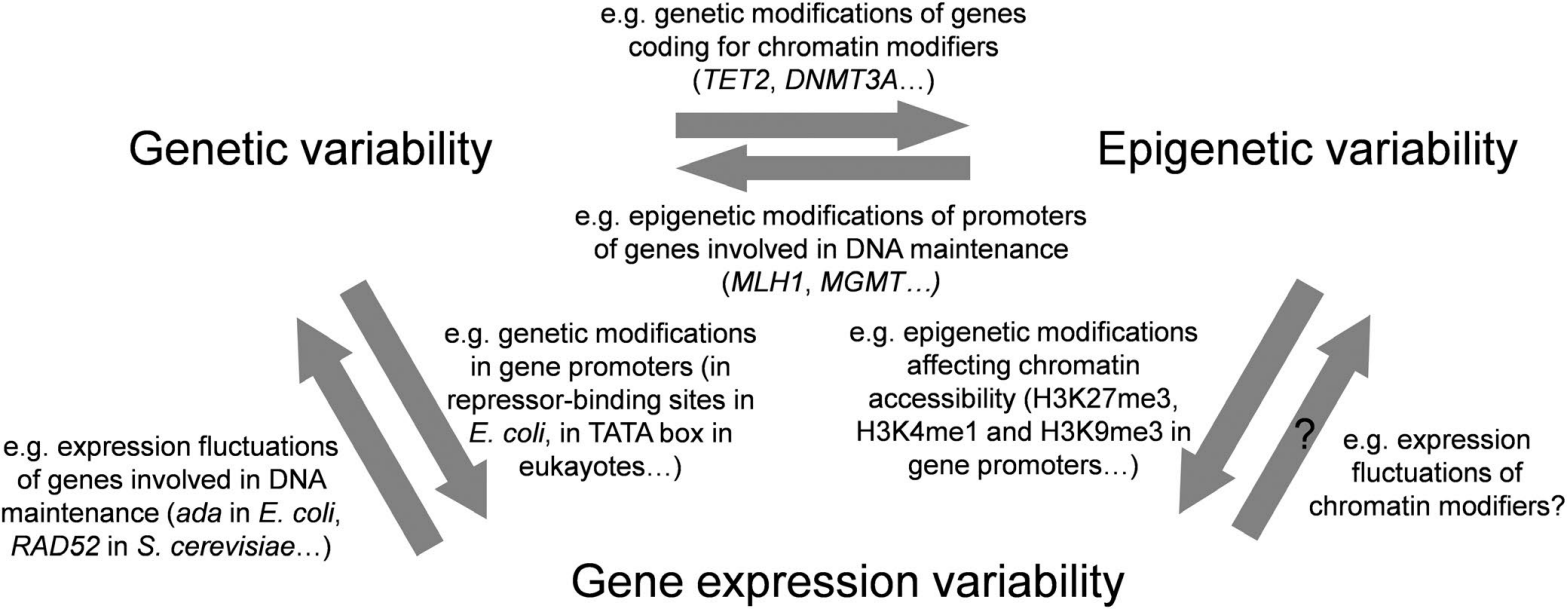
Interplay between genetic, epigenetic, and gene expression variability. The three sources of biological variability studied here are interconnected. Question marks indicate largely unexplored relationships. For each relationship, few examples are given (see the text for details and references)

The interplay between genetic, epigenetic, and gene expression variability is summarized in Figure 1. Comprehensive reviews on the relationships between genetic and epigenetic variability have been provided elsewhere, especially in the onset of cancer hallmarks (Shen & Laird, 2013). For instance, they influence each other in cancer cells through genetic modifications of genes coding for chromatin modifiers on the one hand and epigenetic modifications of promoters of genes involved in genetic stability on the other hand. The present article rather considers the introduction in this interplay of the third category—that is, gene expression variability. Each one of the four arrows connected to gene expression variability (Figure 1) is examined.

Genetic and epigenetic influences on gene expression variability begin to be well characterized (Eling et al., 2019; Sanchez & Golding, 2013), while the reverse relationships are poorly explored. This article aims for the first time at both giving up‐to‐date description of the formers and highlighting recent works investigating the possible consequences of the latters, especially the influence of gene expression variability over epigenetic variability that just starts to be investigated.

## 2 INFLUENCE OF GENETIC VARIABILITY OVER GENE EXPRESSION VARIABILITY

The influence of genetic variability over gene expression variability is by far the most studied relationship among the four considered here. Since the seminal works in the early 2000s on noise in bacteria (Elowitz et al., 2002) and yeast (Blake et al., 2003; Raser & O'Shea, 2004), the genetic determinants and cellular constraints on noisy gene expression were well described (Sanchez & Golding, 2013). Especially, the influence of genetic variations in gene promoters and other *cis*‐elements over the level of noise has been widely studied, especially in eukaryotic cells. Studies on the effect of promoter architecture identified the main genetic determinants that modulate transcriptional burst frequency and size (the average number of mRNAs synthesized during a burst of transcription) and thus intrinsic noise [for a recent update on the concept of transcriptional bursting, see (Tunnacliffe & Chubb, 2020)].

In bacterial cells, it was soon shown that expression noise is affected at both the transcriptional and translational levels, translational bursts having the largest effect on cell‐to‐cell variability (Ozbudak et al., 2002; Thattai & van Oudenaarden, 2001). On the contrary, transcriptional bursting was assumed to be the major determinant in eukaryotic cells (Bar‐Even et al., 2006; Blake et al., 2003; Newman et al., 2006). High expression variability in prokaryotes results predominantly from low transcription and efficient translation (Ozbudak et al., 2002; Thattai & van Oudenaarden, 2001), with the consequence that noise can be reduced by less efficient ribosomal binding sites or mutations in the start codon (Ozbudak et al., 2002). Nevertheless, later works on *Escherichia coli* suggested that transcription and translation contribute to expression variability at approximately the same level in prokaryotes (Guimaraes et al., 2014). This was corroborated by a genome‐wide study showing that half of the *E*.* coli* promoters has significant promoter‐specific levels of noise (Silander et al., 2012). This level can be tuned in these cells by changing promoter‐specific features such as repressor‐binding site sequences (Jones et al., 2014).

In *Saccharomyces cerevisiae*, genes with TATA box‐containing promoters show higher variability than the other genes (Newman et al., 2006; Zaugg & Luscombe, 2012; Zhang et al., 2009) and mutations in TATA box decrease promoter‐mediated expression noise (Blake et al., 2006; Hornung et al., 2012; Raser & O'Shea, 2004). This relationship has also been observed in mammalian genomes (Faure et al., 2017; Larsson et al., 2019; Ochiai et al., 2020; Tantale et al., 2016; Zoller et al., 2015) where genes with TATA elements have larger burst sizes and intrinsic variability compared to genes without such elements (Larsson et al., 2019; Ochiai et al., 2020), even if other works found negligible function of TATA box in regulating expression noise in human embryonic cells (Wu et al., 2017).

Transcription factor (TF)‐binding sites strength, number, and position also have an influence (Sanchez et al., 2013; Sanchez & Golding, 2013): For instance, promoters with more TF‐binding sites exhibit higher expression variability (Sharon et al., 2014; To & Maheshri, 2010), which might due to the stochasticity of TF binding and falling off (Sanchez et al., 2013). Moreover, noise increases when a repressor‐binding site is moved closer to the TATA box within a promoter in yeast (Murphy et al., 2007). Recent works showed that the presence of several transcription regulators in promoters is positively correlated with burst size, while those bound to enhancers are poorly correlated (Ochiai et al., 2020), suggesting that burst size is primarily controlled at the core promoter level. On the contrary, distal enhancers mainly control burst frequency and thus noise (Bartman et al., 2016; Fukaya et al., 2016; Larson et al., 2013; Larsson et al., 2019; Ochiai et al., 2020).

Finally, other effects of local sequence architecture should be highlighted. Especially, transcriptional variability decreases with the number of transcriptional start sites (TSSs) and the presence of CpG islands in gene promoter, TSS, and gene body (Faure et al., 2017). Other works showed that CpG island size is negatively correlated with expression noise (Morgan & Marioni, 2018). Similarly, polynucleosome‐disfavoring sequences in promoters confer lower transcriptional variability (Sharon et al., 2014). Indeed, according to the simplest model of promoter activation with two alternative—OFF (silenced) and ON (initiated)—states (Tunnacliffe & Chubb, 2020), switching between ON and OFF states should reflect chromatin remodeling (Golding & Cox, 2006), with nucleosome‐binding sites generating transient open states leading to transcriptional bursting, which generates more variability in gene expression (Sanchez & Golding, 2013) (see below).

Given these multiple influences of *cis*‐elements, genetic variations are expected to modulate transcriptional variability. For instance, the mutational effects on mean and noise of a large number of natural yeast promoter variants were revealed (Liu et al., 2015; Metzger et al., 2015). Especially, promoter mutations increasing noise appear to be under purifying selection (Metzger et al., 2015). As suggested by previous works on synthetic promoters (Blake et al., 2006), promoter engineering toward such enhancement can be highly beneficial in stressful conditions (Liu et al., 2018). Later works with mutant alleles of the *TDH3* promoter in yeast indeed demonstrated the fitness effects of modifications of gene expression variability through genetic variation (Duveau et al., 2018). The fitness effect of mutations in 33 yeast promoters was also used to more globally study the fitness landscape of mean‐noise expression space (Schmiedel et al., 2019). It revealed that enhanced variability is detrimental for many genes in normal conditions (while this would not be necessarily the case in stressful conditions). It also showed the importance of considering the interplay between genetic and gene expression variability in the understanding of the mechanisms that shape variation in *cis*‐regulatory sequences.

## 3 INFLUENCE OF EPIGENETIC VARIABILITY OVER GENE EXPRESSION VARIABILITY

Epigenetic mechanisms have also strong effects on gene expression variability. Mutations in chromatin remodelers clearly affect this phenomenon in yeast (Weinberger et al., 2012). Among the *cis*‐elements mentioned above, several impact chromatin properties, especially nucleosome occupancy. Promoters with nucleosome‐binding sites alternate between silenced or open states. Their transient opening and reclosing leads to transcriptional bursting events of variable duration and frequency, generating cell‐to‐cell variability in gene expression (Sanchez & Golding, 2013). Especially, nucleosome density around TSSs influences burst frequency (Brown et al., 2013; Dey et al., 2015) and expression noise (Small et al., 2014; Tirosh & Barkai, 2008). This is why promoters with polynucleosome‐disfavoring sequences exhibit less noise: Their presence produces higher burst frequencies and thus leads to lower cell‐to‐cell heterogeneity (Sharon et al., 2014).

First, as high nucleosome occupancies are associated with TATA elements and highly variable genes (Tirosh & Barkai, 2008), the presence of a TATA box is expected to impact expression through the nucleosome architecture favored by this element. Indeed, a qualitative model for nucleosome positioning in yeast showed that nucleosome positioning in TATA‐containing promoters increases transcriptional variability (Zaugg & Luscombe, 2012). Similarly, promoters containing both TATA boxes and nucleosome‐occupied TF‐binding sites exhibit such high expression noise (Field et al., 2008). On the contrary, in human embryonic cells, TSS‐proximal nucleosome occupancy is only weakly correlated with expression variability (Wu et al., 2017).

Epigenetic modifications can also influence cell‐to‐cell expression variability. In gene bodies, DNA methylation at CpG dinucleotides suppresses transcriptional noise (Huh et al., 2013). Histone modifications also impact this phenomenon through chromatin accessibility (Wu et al., 2017), with promoter‐proximal modifications being associated with increased noise (H3K27me3, H3K4me1, and H3K9me3) and modifications in gene bodies being associated with decreased noise (H3K36me3, H3K4me3, and H3K9ac) (Faure et al., 2017). Intriguingly, gene body histone marks determine more gene expression variability than promoter sequence features (Faure et al., 2017). Mechanistically, the promoter histone‐acetylation level directly influences burst frequency for many genes, while burst size is less impacted (Nicolas et al., 2018). H3K27ac and H3K9ac are especially highly correlated with burst frequency (Nicolas et al., 2018).

Previous works showed that some bivalent promoters with both the repressive H3K27me3 mark (catalyzed by the Polycomb Repressive Complex 2 [PRC‐2]) and the activation‐associated H3K4me3 mark especially display high variability in mouse embryonic stem cells that might be caused by switching between the repressed and active states (Kar et al., 2017). In fact, Polycomb could modulate bursting and enhance switching between ON and OFF states in this subset of genes (Kar et al., 2017). Finally, more recent works showed that promoter binding of PRC‐2‐related factors influence bursting and have effects on specific genes (Ochiai et al., 2020). On the other hand, the effects of PRC2‐related factors bound on promoter are opposite from those on gene body (Ochiai et al., 2020).

Single‐cell nucleosome mapping revealed cell‐to‐cell variation in nucleosome occupancy during induction of the *PHO5* gene in yeast, and a small fraction of cells that exhibit nucleosome‐free regions at the promoter even in nonstressed environments (Small et al., 2014), suggesting that nucleosome positioning heterogeneity contributes to gene expression variability independently of the promoter sequence. Indeed, this nucleosome promoter variation was proposed to arise stochastically and to be a source of gene expression variability (Brown & Boeger, 2014). Also, single‐cell chromatin accessibility studies showed a link between accessibility and expression heterogeneity (Buenrostro et al., 2015). Especially, improved accessibility of the promoter for the transcription machinery during initiation thanks to increased eviction or sliding of the nucleosomes probably reduces expression noise during induction (Rawal et al., 2018).

Finally, the chromatin influence on gene expression variability is revealed by various works showing that changing the location of a gene changes its level of noise in *S*.* cerevisiae* (Becskei et al., 2005), *Candida albicans* (Anderson et al., 2014), or chicken cells (Vinuelas et al., 2013). Expression mean and variability are uncorrelated across genomic locations in mammalian cells (Dey et al., 2015). In these cells, genomic locations displaying higher expression noise are associated with more repressed chromatin, thereby indicating that the level of noise is influenced by the chromatin environment (Dey et al., 2015).

## 4 INFLUENCE OF GENE EXPRESSION VARIABILITY OVER GENETIC VARIABILITY

The impact of gene expression variability on cellular response to DNA damage and mutagenesis has been more recently studied, mainly in bacterial cells. These investigations were possible thanks to the development of single‐molecule imaging methods to analyze bacterial DNA–repair processes (Ghodke et al., 2018; Robert et al., 2019; Uphoff & Sherratt, 2017). A pioneering work monitored how expression variation of the Ada protein impacts the response to DNA alkylation damage in *E*.* coli* (Uphoff et al., 2016). The initial expression level of Ada determines the induction times of the damage response at the single‐cell level, producing cell‐to‐cell heterogeneity in DNA repair in the population, especially with cells that do not respond for generations because no Ada proteins are initially expressed (Uphoff et al., 2016). Thus, this subpopulation accumulates foci of the DNA mismatch recognition protein MutS which was used as a marker for labeling nascent mutations (Uphoff et al., 2016). This observation led the authors to conclude that heterogeneity in mutation rate exists at the single‐cell level in *E*.* coli* and that nongenetic variation in protein abundances can lead to genetic heterogeneity. These results were next confirmed in a study showing that the different rates at which the mismatches stochastically arise in single cells depend upon both the preexisting levels of DNA damage‐processing proteins and the cell's ability to up‐regulate its own repair responses (Uphoff, 2018).

Concomitant works finely quantified mutation rates (Robert et al., 2018) and spontaneous DNA replication errors (Woo et al., 2018) in single *E*.* coli* cells, while not connecting it to expression variability of genes involved in DNA transactions. Several molecular processes can be evoked to explain cell‐to‐cell variation in mutagenesis: stochastic events in DNA damage and repair, heterogeneous expression of DNA repair genes or variation in other cellular processes influencing DNA repair such as cell cycle or cell growth rate [for a comprehensive review, see (Vincent & Uphoff, 2020)]. Thus, gene expression variability is only one of the parameters that can generate intercellular variation in mutation rate.

Besides DNA repair activity in bacteria, heterogeneity in homologous recombination (HR) rate in relationship with gene expression variability was also studied with another experimental approach using cell sorting in *S*.* cerevisiae* (Liu et al., 2019). Here, genes impacting HR either directly through their involvement in the pathway (*RAD52*) or indirectly through their involvement in DNA replication (*RAD27*) were fused to a fluorescent marker that allowed to sort cells with extreme expression levels in the population. By using a strain harboring an intrachromosomal HR substrate, it was possible to demonstrate the existence of a high heterogeneity in HR rate among yeast cells, with low Rad52 or Rad27 levels being associated with lower HR rate (Liu et al., 2019). Effects of cell cycle heterogeneity and heterogeneity in DNA damage between the subpopulations were excluded so as to conclude that HR rate heterogeneity is directly caused by gene expression noise. By sorting more subpopulations than the two extreme ones, Rad27 levels were shown to nonlinearly scale with HR activity. This observation has important consequences because it means that the total amount of HR does not depend only on the averaged Rad27 expression: Doubling the mean Rad27 expression would not lead to a doubling of HR rate, and slight modifications of its mean expression level could generate high variation in HR activity.

Thus, subpopulations with increased mutation or HR rates are expected to improve whole population adaptability in changing environments because they constitute a reservoir of increased genetic variability, with important evolutionary consequences (Matic, 2019; Woo et al., 2018). The presence of subpopulations of cells with enhanced mutagenesis could be particularly important when several mutations are required for adaptation to new environments (Alexander et al., 2017). Gene expression variability has a clear role in the appearance of this heterogeneity and modifying noise in the expression of genes involved in the DNA transaction processes could allow tuning the mutation and/or recombination rates and have consequences in terms of evolvability in fluctuating environments (Capp, 2010).

## 5 INFLUENCE OF GENE EXPRESSION VARIABILITY OVER EPIGENETIC VARIABILITY

Almost nothing is known about the influence of gene expression variability over epigenetic variability. The possible influence of noise in the expression of epigenetic regulators on chromatin modification activity in *S*.* cerevisiae* was recently explored by analyzing the well‐known histone modifier of the Sirtuin family Sir2 and epigenetic silencing at subtelomeres (Liu et al., 2020). Like for *RAD52* and *RAD27* in the HR context, *SIR2* was fused to a fluorescent marker allowing to sort cells with extreme expression levels in a strain containing a subtelomeric *URA3* silencing substrate. No difference in silencing activity was observed, probably because Sir2 is not limiting for silencing subtelomeric *URA3* (Liu et al., 2020). Nevertheless, this might not be true for other reporter genes and other genomic locations. Finally, other chromatin remodelers and epigenetic events should be tested.

## 6 APPLIED QUESTIONS IN THE CONTEXT OF ONCOGENESIS

Neoplasms are characterized by a global increase in cellular stochasticity and intra‐tumoral heterogeneity (ITH), with higher epigenetic and gene expression variability compared to normal cells (Jenkinson et al., 2017). Together with their well‐known genetic instability, these features of cancer cells constitute an ideal model to decipher their interplay. The level of genetic ITH was found to be positively correlated with the level of epigenetic ITH in leukemia (Landau et al., 2014; Oakes et al., 2014) or aggressive prostate cancer (Brocks et al., 2014), among others (Mazor et al., 2016). In addition to this correlation, promoters with high methylation ITH in leukemia were also associated with high cell‐to‐cell expression heterogeneity of the corresponding gene (Landau et al., 2014), suggesting that genetic, epigenetic, and gene expression ITHs are mostly correlated. They probably contribute concomitantly to cancer cell evolvability and to the increase of phenotypic diversity in cancer cell populations during progression. Their interplay in oncogenesis deserves to be investigated in an evolutionary perspective, especially how they dynamically interact and change when the rate of appearance of genetic, epigenetic, or gene expression diversity is modified.

Therefore, it should be interesting to see how the two other types of variability dynamically evolve when the third is experimentally enhanced. The approach that consists in studying cancerous phenotypes arising upon artificially imposed gene expression noise, using noise‐controlling genetic devices, methods, or chemicals (Guinn et al., 2020), is certainly relevant in this context for studying the influence of gene expression variability over the others. Would cell populations follow distinct evolutionary trajectories mainly based on the one that has been increased, or would they converge in all cases toward similar states relying on the same mixture of genetic, epigenetic, and gene expression variability? On the contrary, would targeting one of them be somehow compensated by an increase in the other types of variability, and which targeting would be more efficient in decreasing cellular stochasticity and acquiring phenotypic stability? Answering these questions might open new therapeutic perspectives on rational combinations of genetic‐, epigenetic‐, and/or gene expression‐based strategies able to counteract the still underunderstood power of cellular evolvability (Payne & Wagner, 2019).

Finally, studying this interplay in oncogenesis is especially interesting in the context of drug resistance. The interplay between genetic and nongenetic phenomena has already been highlighted in the appearance of resistant cells (Bell & Gilan, 2020; Salgia & Kulkarni, 2018). A recent work explicitly studied the complex interplay among genetic, epigenetic, and stochastic sources of ITH in the context of drug resistance and showed that in almost all clonal sublines, drug‐response variability is due to epigenetic rather than genetic differences (Hayford et al., 2020). Nongenetic heterogeneity in diverse epigenetic and gene expression processes now appears to be a major contributor in the appearance of persistent cancer cells, which constitute a rare subpopulation that can survive cancer drug treatment and constitute a major cause of treatment failure (Shen et al., 2020). For instance, the rare and transient transcription of a number of resistance markers at high levels in a very small percentage of single melanoma cells is at the origin of a resistance phenomenon due to transcriptional variability, which is followed by epigenetic “reprogramming” in these cells converting this transient transcriptional state to a stably resistant state (Shaffer et al., 2017). These groups of genes co‐fluctuate in “coordinated rare‐cell expression programs” and are heritable for several generations but ultimately transient (Shaffer et al., 2020). A prominent role of gene expression variability in the emergence of resistant cells was also found among estrogen receptor‐positive breast cancers (Hinohara et al., 2018). When gene expression stochasticity and transcriptomic and phenotypic heterogeneity were decreased through the inhibition of the activity of members of the KDM5 demethylase family, resistance to endocrine therapies was reduced because less cells acquire resistance (Hinohara et al., 2018).

Cells surviving through nongenetic mechanisms can then give rise to genetic resistance upon continuous anticancer treatment (Hata et al., 2016; Ramirez et al., 2016; S. Shen et al., 2019). Thus, tuning these nongenetic mechanisms and the level of diversity that they generate could impact the evolution of drug resistance in mammalian cells. Such a modulation of gene expression variability was recently used to study whether high or low expression noise of specific resistance proteins and network circuits can generate stable resistance by acquisition of mutations within the circuits (Farquhar et al., 2019). Only cells with the low noise gene circuit mutated to stably adapt, showing the relevance of studying this interplay and its implications in cancer treatments with known regulatory mechanisms of resistance (Farquhar et al., 2019). The works mentioned above showing that higher noise can promote survival for cell populations via mutagenesis could appear contradictory with the Farquhar et al's study, but the low versus high noise circuit in the latter drives the expression of the gene that confers resistance, not directly genes involved in DNA maintenance as in the formers. Thus, adaptation by mutations would be favored when noise in the expression of genes involved in DNA maintenance is higher and when noise in the expression of genes involved in therapeutic resistance is lower. Studies on the interplay between genetic, epigenetic, and expression variability, and the role of noise in evolutionary trajectories, could be particularly challenging in cancer cell populations because of the many disrupted regulatory levels and the high levels of instability that characterize cancer cells, but the importance of these phenomena in this context also shows their high applicability.

## 7 CONCLUSION

The interplay between genetic, epigenetic, and gene expression variability adds layers of complexity in the generation of phenotypic variation. These three sources of biological variability are generally considered independently in their relationship with phenotypic variation while they appear to be intrinsically interconnected and influence it in combination. Future works will be needed to decipher still largely unexplored influences, especially how epigenetic mechanisms might be impacted by expression variability of chromatin remodelers, and to apply these concepts in the context of complex and fluctuating environments such as the ones found in oncogenesis.

## CONFLICT OF INTEREST

None declared.

## Data Availability

Data sharing is not applicable to this article as no new data were created or analyzed in this study.
